# Inhibitory Compounds Targeting Plasmodium falciparum Gyrase B

**DOI:** 10.1128/AAC.00267-21

**Published:** 2021-09-17

**Authors:** Zuzanna Pakosz, Ting-Yu Lin, Elizabeth Michalczyk, Soshichiro Nagano, Jonathan Gardiner Heddle

**Affiliations:** a Malopolska Centre of Biotechnology, Krakow, Poland; b Postgraduate School of Molecular Medicine, Medical University of Warsaw, Warsaw, Poland; c Heddle Initiative Research Unit, RIKEN, Wako, Saitama, Japan; d Department of Cell Biochemistry, Faculty of Biochemistry, Biophysics and Biotechnology, Jagiellonian University, Krakow, Poland

**Keywords:** DNA gyrase, antimalarial agents, apicomplexan parasites, apicoplast, fluoroquinolones, purpurogallin, supercoiling, topoisomerases

## Abstract

Malaria persists as a major health problem due to the spread of drug resistance and the lack of effective vaccines. DNA gyrase is a well-validated and extremely effective therapeutic target in bacteria, and it is also known to be present in the apicoplast of malarial species, including Plasmodium falciparum. This raises the possibility that it could be a useful target for novel antimalarials. To date, characterization and screening of this gyrase have been hampered by difficulties in cloning and purification of the GyrA subunit, which is necessary together with GyrB for reconstitution of the holoenzyme. To overcome this, we employed a library of compounds with specificity for P. falciparum GyrB and assessed them in activity tests utilizing P. falciparum GyrB together with Escherichia coli GyrA to reconstitute a functional hybrid enzyme. Two inhibitory compounds were identified that preferentially inhibited the supercoiling activity of the hybrid enzyme over the E. coli enzyme. Of these, purpurogallin (PPG) was found to disrupt DNA binding to the hybrid gyrase complex and thus reduce the DNA-induced ATP hydrolysis of the enzyme. Binding studies indicated that PPG showed higher-affinity binding to P. falciparum GyrB than to the E. coli protein. We suggest that PPG achieves its inhibitory effect on gyrase through interaction with P. falciparum GyrB leading to disruption of DNA binding and, consequently, reduction of DNA-induced ATPase activity. The compound also showed an inhibitory effect against the malaria parasite *in vitro* and may be of interest for further development as an antimalarial agent.

## INTRODUCTION

DNA gyrase is a type II topoisomerase that performs essential topological operations on DNA and has the unique ability to carry out negative supercoiling by using energy provided by ATP hydrolysis (1). In addition, gyrase is a well-known, effective, and widely used antibacterial drug target (2, 3). Along with topoisomerase IV, it is the target of the broad-spectrum fluoroquinolone class of antibacterials, which are used to treat a variety of infections, most notably those of the urinary tract. Its attractiveness as a drug target is increased by the lack of a gyrase enzyme in human cells.

**FIG 1:**
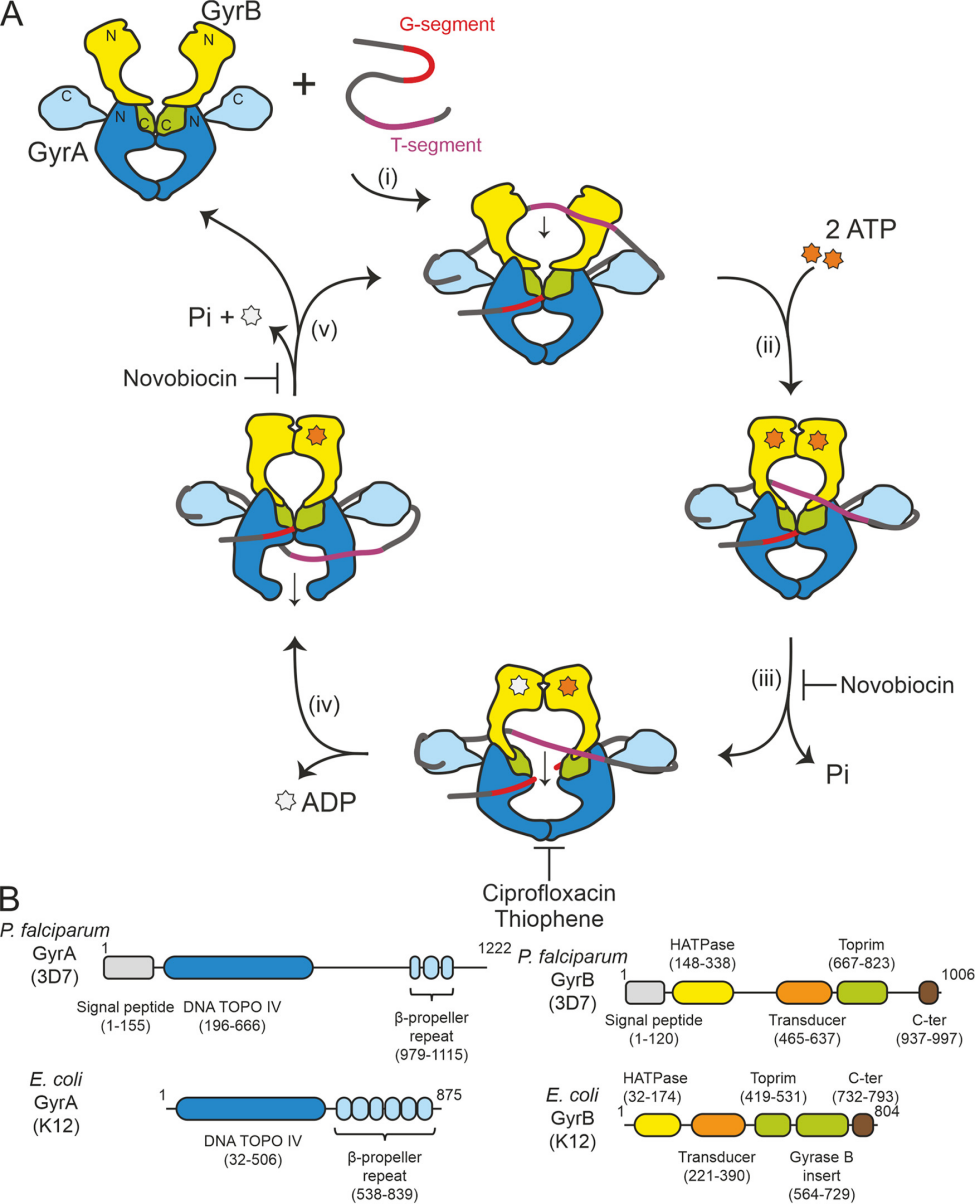
Overview of the bacterial gyrase-mediated DNA negative supercoiling catalytic cycle and a schematic showing the domain organization of E. coli and P. falciparum gyrases. (A) The *Ec*Gyr-mediated DNA supercoiling cycle. (i) DNA binds to *Ec*Gyr with *Ec*B capturing the T-strand. (ii) ATP binding to *Ec*B. (iii) ATP hydrolysis by *Ec*B to facilitate the strand passing. Novobiocin inhibits gyrase by competing for binding with ATP. (iv) DNA religation. This step is inhibited by gyrase poisons (e.g., CIP and thiophene), both of which achieve stabilization of the gyrase-cleaved DNA complex. (v) ATP hydrolysis for structural rearrangement and subsequently release of the DNA substrate. (B) Illustration of gyrase domains. Domain information was retrieved from Pfam (45). It has previously been noted (21, 31) that Pfam predicts only three blades in *Pf*GyrA_CTD, but at least two further putative blades have been identified (31). The domains and their sizes are labeled accordingly.

Gyrase is composed of two subunits, GyrA and GyrB, with the functional enzyme being an A_2_B_2_ tetramer. Its mechanism of action and the relationship between structure and function have been well characterized (2) (Fig. 1A). Gyrase is thought to bind to a section of DNA known as the G-segment, which binds across the GyrA dimer interface (the saddle region). The G-segment is cleaved across both strands via formation of a phosphotyrosine bond with the active site tyrosines in the GyrA dimer. For negative supercoiling to occur, the DNA must be wrapped around gyrase with positive handedness, and this wrap is achieved by the C-terminal domains of GyrA which form a six-bladed β-propeller. Another section of the same double-stranded DNA (dsDNA), the “T-segment,” is then captured by the N terminus of GyrB (the “DNA clamp,” which dimerizes upon binding of ATP), passed through the break generated in the G-segment, and finally is released through the “bottom” gate in GyrA.

Bacterial gyrase is the target of a number of antibacterial drugs (4). Most notable are the aminocoumarins and the fluoroquinolones (5). The latter is an extremely important class of drugs that function by binding to the gyrase-DNA complex at the stage where both DNA strands are cleaved (6), a mechanism shared by the thiophenes. These compounds allosterically stabilize the cleavage complex through binding to either one or two DNA strands (7). Stabilization of the cleavage complex eventually results in fragmentation of the bacterial genome and cell death. Aminocoumarins, such as novobiocin, function differently in that they compete with ATP for binding to the ATP binding site in the N-terminal region of GyrB (2, 8).

Gyrase was initially discovered as a bacterial enzyme (9) but later confirmed as being present in some plants (10–12), *Plasmodium* spp., and other members of the Apicomplexa. The genes of apicomplexan protozoan gyrase are contained in the nuclear genome, but the functional DNA gyrase is present in a plastid called the apicoplast (13, 14). The apicoplast is a four-membraned organelle found in some protozoa (15). It is a result of secondary endosymbiosis wherein a prokaryotic cyanobacterium was incorporated into a eukaryote and subsequently engulfed by another eukaryote (16). It is indispensable due to its important role in biosynthesis of fatty acid, isoprenoids, iron-sulfur clusters, and heme (17). Due to its bacterial origin, it retains a circular genomic DNA that requires the nucleus-encoded gyrase to resolve topological challenges (18). To date, little characterization of apicoplast gyrases has been carried out (19, 20). Bioinformatics analyses suggest that Plasmodium falciparum GyrA (*Pf*A) and P. falciparum GyrB (*Pf*B) retain the expected domain structure as well as the conserved catalytic residues (18, 21). Interestingly structure prediction tools predict fewer than the expected number of propellers in the β-propeller domain. These gyrases are considerably larger than other characterized gyrases, containing stretches of additional unannotated sequences, the functions of which are currently unclear (21) (Fig. 1B; see Fig. S1A and B in the supplemental material).

Given the effectiveness of gyrase as a lethal bacterial target, its presence in *Plasmodium* spp. immediately raises the prospect of using apicoplast gyrase as a novel antimalarial target as well as in toxoplasmosis given that gyrase is also necessary for apicoplast genome replication and parasite growth in Toxoplasma gondii (22). The pattern of conservation between apicoplast and well-characterized prokaryotic gyrases suggests that binding sites for gyrase-targeting antibacterials are likely to remain highly similar. An apicoplast gyrase-targeting agent should have few side effects given that gyrase does not occur in humans (18). Indeed, antibacterials have been used against the parasite, including gyrase-targeting ciprofloxacin (CIP), which results in a “delayed death” phenomenon as a result of arresting of growth in the second asexual cycle (23). Among fluoroquinolones tested, CIP has been reported as having best antimalaria activity but had off-target (i.e., non-gyrase/topoisomerase IV) toxicity, which may have been partially responsible for the killing effect observed (24–26). In contrast, other reports have shown that fluoroquinolones do not appear to be particularly effective antimalarial agents against uncomplicated malaria and suggested they should be used together with other standard antimalarials (27, 28). Furthermore, targeting malaria with antibacterials such as gyrase poisons may not be wise, as it would have the potential to accelerate the spread of antibacterial resistance (29).

For these reasons, we have attempted to discover new molecules that specifically inhibit malarial apicoplast gyrase but not bacterial gyrase. As P. falciparum causes the most severe outcomes among all *Plasmodium* species (24, 30), we chose to work with P. falciparum gyrase (*Pf*Gyr) proteins. To test for inhibitors, it is necessary to produce purified *Pf*Gyr for *in vitro* experiments. Unfortunately, it has not yet proven possible to express and purify functional *Pf*A (31). However, expression and purification of *Pf*B have been reported (20, 32). *Pf*B is known to be able to form an active, supercoiling-competent gyrase complex with Escherichia coli GyrA (*Ec*A), while its intrinsic ATPase activity can be induced by the addition of DNA (20).

We have cloned and purified the recombinantly expressed *Pf*B and reconstituted a hybrid gyrase by combining it with *Ec*A (*Ec*A_2_*Pf*B_2_). This was tested against a chemical library consisting of 98 compounds selected from a screen for molecules that bind to *Pf*B but not *Ec*B. Those hit compounds were then characterized in further functional assays, including ATPase activity, supercoiling, and DNA cleavage activity. This has resulted in the isolation of two candidates. Of these, purpurogallin (PPG) {3,9,10,11-tetrahydroxybicyclo[5.4.0]undeca-1(7),3,5,8,10-pentaen-2-one} was found to be the most promising and was characterized further.

## RESULTS

### P. falciparum gyrase production and purification.

To fully characterize *Pf*Gyr, it would be necessary to reconstitute the holoenzyme. However, attempts to produce the full *Pf*Gyr complex were not successful due to difficulties in *Pf*A production, even after extensive trials, consistent with the previous report (20). In contrast, full-length (without the predicted signal peptide) mature *Pf*B production was achieved, as reported in previous studies (Fig. S1C). *Pf*B exists as a homodimer and can assemble a functional gyrase complex with *Ec*A (20). In addition, this hybrid complex is sensitive to known bacterial gyrase inhibitors, including CIP and novobiocin, although sensitivity to the former is less than that seen for *Ec*Gyr (Fig. S1D and E).

### Screening reveals small compounds able to specifically inhibit supercoiling activity of *Pf*B-containing gyrases.

**FIG 2:**
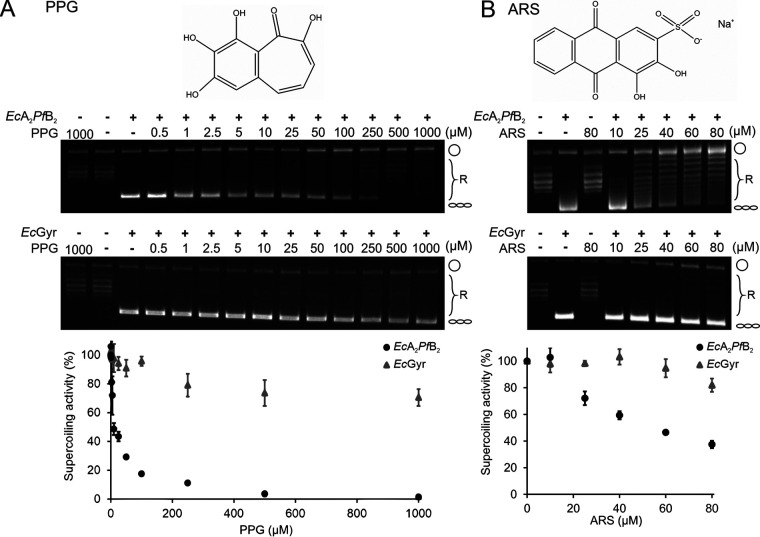
PPG and ARS preferentially inhibit *Ec*A_2_*Pf*B_2_ DNA supercoiling. (A and B) Chemical structures of PPG and ARS are shown (top). Representative gel images of DNA supercoiling by hybrid gyrase or *Ec*Gyr against PPG or ARS (middle). Concentrations of inhibitors are as labeled above the lanes. Cartoons to the right of gels indicate the topological state of the corresponding bands on the gels, being open circular (circle), relaxed (R), or negatively supercoiled (helix). Plots summarizing multiple experiments (*n* = 3), including hybrid gyrase (black circles) and *Ec*Gyr (gray triangles), are shown below the relevant gels, and the standard deviation (SD) is plotted on the graphs. PPG and ARS were dissolved in DMSO, and the final DMSO concentration in the assay was 1%.

We used a chemical library consisting of 98 compounds selected based on preferential binding to *Pf*B over the bacterial *Ec*B protein. We further screened this library against the supercoiling activity of the hybrid enzyme (see Fig. S2A in the supplemental material). Two compounds showing significant inhibition were identified: PPG and alizarin red S (ARS) (1,2-dihydroxy-3-(oxysulfonyl)-9,10-anthracenedione) (Fig. S2A in the supplemental material; lane A10 is PPG, and lane B6 is ARS). PPG specifically inhibited the supercoiling activity of the hybrid gyrase (50% inhibitory concentration [IC_50_], 9.56 ± 1.94 μM) but not that of bacterial gyrase (IC_50_, >1,000 μM) (Fig. 2A and Table 1). Similarly, ARS also preferentially inhibited the hybrid gyrase (IC_50_, 55.59 ± 6.74 μM) (Fig. 2B and Table 1). PPG, compared to ARS, showed better efficacy in inhibiting the hybrid gyrase supercoiling ability. We then focused on characterizing PPG-mediated inhibition both *in vivo* and *in vitro* in this study.

**TABLE 1:** IC_50_ of compounds in supercoiling inhibitory activity

Antimalarial drug	IC_50_ (μM) for target:	
	*Ec*Gyr	*Ec*A_2_*Pf*B_2_
CIP	0.91 ± 0.33	7.06 ± 0.76
Novobiocin	1.04 ± 0.22	1.83 ± 0.39
PPG	>1,000	9.56 ± 1.94
ARS	>100	55.59 ± 6.74

### PPG decreases the DNA-enhanced ATP hydrolysis rate of *Pf*B.

**FIG 3:**
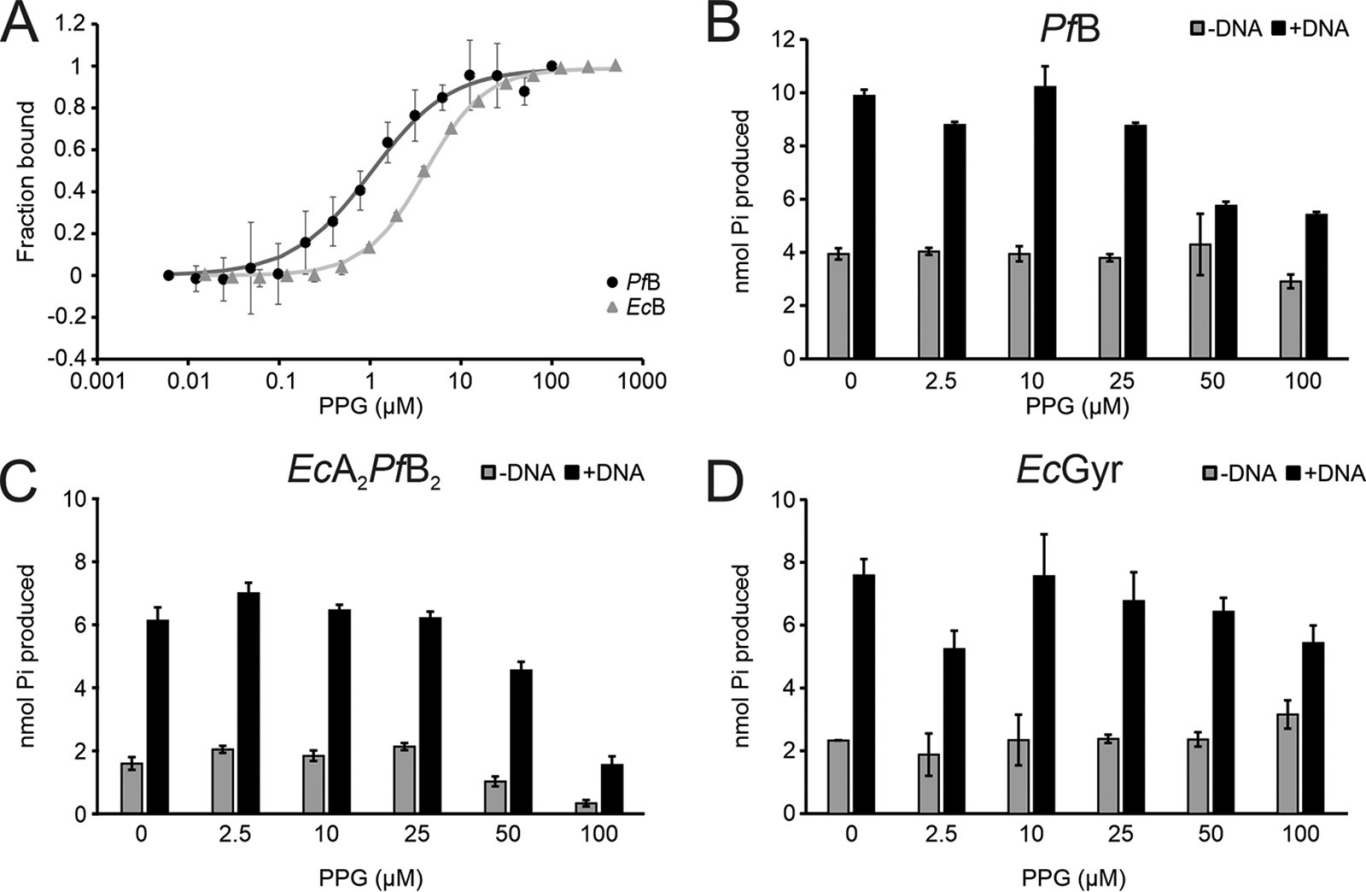
PPG interaction with GyrB subunits and its influence on DNA-induced ATP hydrolysis of *Pf*B, the hybrid complex, and *Ec*Gyr. (A) PPG binds to *Pf*B preferentially in comparison to *Ec*B. Fluorescently labeled proteins were incubated with titrated PPG, and then samples were measured using MST. The curve presented on the graph represents fitting with a 1:1 binding curve. (B to D) PPG influence on DNA-induced ATP hydrolysis activity for *Pf*B (B), *Ec*A_2_*Pf*B_2_ (C), or *Ec*Gyr (D). The final DMSO concentration was 1%. *n* = 3. The standard error (SE) is plotted in the graph.

Given that PPG was identified based on binding to an *Ec*A_2_*Pf*B_2_ complex in preference to a *Ec*Gyr complex, we hypothesized *Pf*B as the most likely binding site. We tested PPG binding to *Pf*B and *Ec*B using microscale thermophoresis (MST). MST profiles showed that PPG binding to both GyrB proteins followed a sigmoidal shape. The curve for *Pf*B (dissociation constant [*K_d_*], ∼1.03 ± 0.11 μM) was steeper than that for *Ec*B (*K_d_*, ∼4.54 ± 0.39 μM), which indicates tighter binding in the case of *Pf*B (Fig. 3A). Because the MST curves fitted a sigmoidal 1:1 binding model, it also suggests that one PPG is bound per monomer (two PPGs per *Pf*B dimer). Next, we investigated the effects of PPG’s interaction with *Pf*B. It has been reported that *Pf*B alone has intrinsic ATP hydrolysis activity, and this activity can be enhanced by the presence of DNA (20). We performed ATP hydrolysis assays and determined the effect of PPG on *Pf*B activity. As expected, the intrinsic ATP hydrolysis activity of *Pf*B, in the absence of the A subunit, was increased around 3-fold by the addition of DNA (Fig. 3B). The addition of PPG decreased this DNA-enhanced ATP hydrolysis of *Pf*B in a dose-dependent manner, while the intrinsic ATPase activity remained unchanged. We further observed that PPG treatment also inhibited the DNA-induced ATPase activity of *Ec*A_2_*Pf*B_2_, although this was more modest (Fig. 3C). On the other hand, both intrinsic ATP hydrolysis and enhanced ATP hydrolysis by *Ec*B in the complex with *Ec*A were little affected by the presence of PPG. Overall, these results suggest that the PPG mechanism of action is distinct from those of competitive ATP binders, such as novobiocin, and likely acts via interaction with a different site on GyrB.

### PPG decreases DNA binding to *Pf*B.

**FIG 4:**
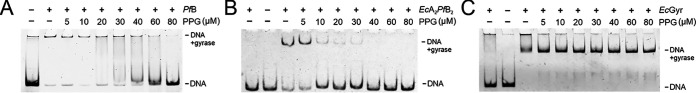
PPG specifically abolished DNA binding to *Ec*A_2_*Pf*B_2_ and *Pf*B. (A to C) EMSA showing the effect of increasing amounts of PPG in retarded migration of the DNA band caused by binding to gyrase proteins. Results are shown for *Pf*B (A), *Ec*A_2_*Pf*B_2_ (B), or *Ec*Gyr (C). DNA (147 bp) was stained with SYBR gold for visualization. The final DMSO concentration was 1%.

The effect of PPG on DNA-stimulated ATPase activities of *Pf*B and *Ec*A_2_*Pf*B_2_ suggested a possible mechanism whereby DNA binding is inhibited. To test that hypothesis, gel-based DNA binding assays were performed in which the binding of *Pf*B and DNA was observed as a shift of the DNA band. In this case, the shifted *Pf*B-DNA complex was not able to enter the gel even after extensive optimization trials—possibly due to the overall charge of the complex. In contrast, the DNA-*Ec*A_2_*Pf*B_2_ complex was visible as a shifted band on the gel. To investigate the effect of PPG on the DNA binding ability of proteins, PPG was added to the reaction with *Ec*A_2_*Pf*B_2_ and *Pf*B alone (Fig. 4). We found that formation of the DNA-*Pf*B complex was inhibited at approximately 20 μM PPG and was observed as the appearance of the free DNA fragment in the gel (Fig. 4A). Furthermore, the DNA-*Ec*A_2_*Pf*B_2_ complex was similarly disrupted at 10 μM PPG, and the interaction was completely abolished at 40 μM PPG (Fig. 4B). In contrast, the DNA-*Ec*Gyr complex remained intact in the presence of PPG (Fig. 4C). In addition, we did not observe any linear DNA formation by PPG treatment using the DNA cleavage assay. This observation suggests that PPG inhibits interactions with DNA either directly or via GyrA-GyrB complex destabilization rather than stabilizing the protein cleaved-DNA complex (Fig. S2B).

### PPG inhibits growth of P. falciparum.

We further investigated the effect of PPG on P. falciparum survival rates *in vitro*. PPG was found to negatively affect the growth of P. falciparum (3D7) *in vitro*. The growth-inhibitory IC_50_ of PPG on P. falciparum was 92 ± 16 μM after 96 h of treatment (Table 2). This IC_50_ is comparable to those of other tested compounds, such as quinolones or ENT1 inhibitors (25, 33–35), which range from 5 to 100 μM. Although the efficacy of PPG at inhibiting P. falciparum growth is not as high, the fact that it is specific to the P. falciparum protein suggests it may offer a useful basis for further development of P. falciparum or apicomplexan-specific gyrase inhibitors.

**TABLE 2:** IC_50_ of compounds for inhibition of P. falciparum growth *in vitro*

Antimalaria drug(s)	IC_50_ in P. falciparum 3D7	Proposed target	Reference(s)
PPG	92 ± 16 μM	*Pf*Gyr	This study
CIP	3 ± 0.5 μM	*Pf*Gyr and off-target effect	25, 33
Quinolines	20–100 μM	Gyrase poisoning	25
ENT1 inhibitors	5–50 μM	*Pf*ENT1	34
Acriflavine	5 mg/kg	DNA replication foci in parasite/indirect decrease in *Pf*Gyr activity by interaction with DNA substrate	35

## DISCUSSION

The discovery of new drugs effective in treating malaria is an important ongoing challenge. P. falciparum DNA gyrase offers a new potential target given the likelihood that its inhibition will have a negative effect on parasite growth (25). Screening and testing of potential *Pf*Gyr-specific inhibitors would ideally be carried out against the native A_2_B_2_ complex. However, as noted, full-length *Pf*A production has not proven possible to date. Similar problems have been observed for the other characterized apicomplexan gyrase from T. gondii, where only minimal amounts of GyrA could be purified and tested (19). It is notable that in both cases, the GyrA proteins in question are thought to contain significant stretches of additional amino acids compared to other known GyrAs, and these are predicted to be flexible, hinting at misfolding as a possible cause of the observed reduced expression (21). Others have shown that truncated forms of *Pf*A (amino acids [aa] 163 to 540, lacking the C-terminal DNA-wrapping domains) can form complexes with cognate, full-length *Pf*B. The resulting complexes were shown incapable of carrying out supercoiling but were able to form cleavage complexes in the presence of both calcium and fluoroquinolone (20). In our hands, however, we were unable to recapitulate the previously observed cleavage results.

We have noticed that *Pf*B can support *Ec*A to carry out supercoiling reactions. Interestingly supercoiling by the hybrid enzyme was shown to be approximately 10-fold less sensitive to CIP than the E. coli gyrase (Fig. S1E). While the majority of fluoroquinolone resistance mutations in gyrase map to the region of GyrA close to the catalytic tyrosine, some are known to map to GyrB (36) in the Toprim domain. Notably, *Pf*B has an approximately 45-aa insert close to this region of the Toprim domain (32), raising the possibility that this may be responsible for additional interactions and/or structural features that have an inhibitory effect on fluoroquinolone binding.

In this work, we have shown that PPG has an inhibitory effect specific to *Ec*A_2_*Pf*B_2_, in contrast to *Ec*Gyr. This was demonstrated in decreased supercoiling activity and decreased DNA binding by the hybrid enzyme in the presence of PPG. *Pf*B has a unique feature in that its ATP hydrolysis can be enhanced by DNA binding (20). ATPase results with *Pf*B were consistent with PPG decreasing binding to DNA as the DNA-stimulated activity was reduced in the presence of PPG, while DNA-independent activity was largely unaffected (Fig. 3B). The effect on the DNA-stimulated ATPase activity of *Ec*A_2_*Pf*B_2_ was less significant than on *Pf*B and only slightly greater than on *Ec*Gyr (Fig. 3C and D). Interestingly, in the context of the *Ec*A_2_*Pf*B_2_ complex, PPG decreased the ATPase activity in both the absence and presence of DNA (Fig. 3C). *Pf*B is substantially larger than the prokaryotic homologs and includes significant extra sequences in the Toprim domain (21). It is quite possible that in the context of the hybrid enzyme, the bacterial GyrA interacts with some of these unique features in *Pf*B either to mildly stimulate ATPase activity (and this stimulation is lost through conformational change upon high-concentration PPG treatment) or, conversely, that PPG causes a conformational change in *Pf*B that acts to inhibit intrinsic ATPase activity. In contrast, *Ec*A_2_*Pf*B_2_ binding to DNA appeared to show a greater apparent sensitivity to PPG (complete loss of binding at 5 to 10 μM PPG, while DNA-stimulated ATPase is only significantly reduced at 25 to 50 μM PPG). This likely reflects the lower-affinity binding of the 147-bp DNA used in the electrophoretic mobility shift assays (EMSAs), which is insufficient for full node formation (37, 38).

How PPG achieves inhibition of DNA binding is still unclear. Our MST results and ATPase results demonstrate that the PPG is capable of achieving binding and ATPase inhibition through interaction with *Pf*B alone in the absence of GyrA. This suggests the possibility of a PPG binding pocket unique to *Pf*B that is able to interfere with DNA binding. As already mentioned, *Pf*B has significant additional stretches of amino acids compared to bacterial homologs. These extra regions may regulate some functions of the enzyme, providing a target for inhibitors that is not present in the prokaryotic enzyme. One possible mechanism whereby inhibition by PPG could be achieved would be for the binding of PPG to *Pf*B to destabilize the GyrA-GyrB interface. Thus, it could in turn lead to the observed effect on DNA binding. To further understand PPG’s action on *Pf*B, we have tried extensive cocrystallization trials of *Pf*B and PPG but have been unable to obtain any crystals to date.

Tests of the effect of PPG on P. falciparum growth showed moderate inhibitory efficacy and raise the question of whether the observed inhibition *in vivo* is due to the observed *in vitro* effects on DNA gyrase and will require significant future work to answer. As PPG does not appear to act as a gyrase poison, it is unlikely to be as potent against P. falciparum as poisons such as fluoroquinolones are against many prokaryotic gyrases. However, it is interesting to note that even moderately effective inhibitors may have a role as part of artemisinin-based combination therapies when combined with artemisinin (39, 40). Clearly, targeting enzymes within the apicoplast represents a challenge given the large number of membranes any therapeutic would have to pass through in order to reach the target. This may require the development of appropriate nanoparticle delivery systems (41, 42).

## MATERIALS AND METHODS

### Protein production and purification.

The full-length *Pf*B open reading frame (ORF) (3D7; accession no. Q8I528) was synthesized as a codon-optimized sequence for use in E. coli expression systems. The N-terminal fragment (aa 1 to 120) was omitted when subcloning to the expression vectors as it is predicted to be a signaling peptide and not to be present in mature, functional forms of *Pf*B (31). The mature *Pf*B ORF (aa 121 to 1006) was amplified and cloned to a pET28a+ vector (Genscript, Inc., USA) using NdeI and BamHI restriction enzymes. The plasmid was transformed to BL21(DE3) Gold competent cells (Agilent) and cultured in LB medium at 37°C. When the optical density at 600 nm (OD_600_) reached 0.6, 0.25 mM isopropyl-β-d-thiogalactopyranoside (IPTG) was added to induce protein expression, and cells were cultured overnight at 18°C with shaking. Cells were collected by centrifugation.

Purification of gyrase proteins was carried out using published methods as a guide (43). For *Pf*B protein, the cell pellet was suspended in lysis buffer containing 50 mM Tris-HCl (pH 7.5), 150 mM NaCl, 20 mM imidazole, 10% glycerol, protease inhibitor cocktail (Pierce EDTA-free protease inhibitor tablets; Thermo), and 0.1% Triton X-100. Cells were further lysed by sonication, and the supernatant was separated from insoluble pellet by centrifugation at 50,000 × *g* for 30 min at 4°C. The clear supernatant was loaded onto a HisTrap column (5 ml; GE Healthcare), and bound proteins were eluted in the lysis buffer containing 250 mM imidazole. The eluted fractions were desalted on desalting columns (GE Healthcare) and loaded onto a Mono Q 16/10 ion-exchange column (GE Healthcare) which was equilibrated in TGED buffer (50 mM Tris-HCl [pH 7.5], 1 mM EDTA, 2 mM dithiothreitol [DTT], 10% glycerol). Protein was eluted using a linear gradient of 0.1 to 1 M NaCl gradient in TGED buffer. The pooled protein fractions were concentrated and loaded onto a Superdex 200 16/600 (GE Healthcare) column equilibrated in TGED for gel filtration. The protein was collected, concentrated, snap-frozen in liquid nitrogen, and stored at −80°C.

### DNA supercoiling.

A supercoiling assay was utilized to test a library of compounds previously selected for binding to *Ec*A_2_*Pf*B_2_ but not *Ec*Gyr. The library was a kind gift from Yasumitsu Kondoh, RIKEN. The *Ec*Gyr complex was used as a control allowing selection for specific compounds that target *Pf*B. Supercoiling assays were carried out in a buffer consisting of 35 mM Tris-HCl (pH 7.5), 24 mM KCl, 4 mM MgCl_2_, 2 mM DTT, 1.8 mM spermidine, 1 mM ATP, 6.5% (wt/vol) glycerol, and 0.1 mg/ml bovine serum albumin (BSA). The hybrid (*Ec*A_2_*Pf*B_2_) complex or *Ec*Gyr complex was formed in the storage buffer (50 mM Tris-HCl [pH 7.5], 100 mM KCl, 10% [wt/vol] glycerol, 1 mM EDTA, 2 mM DTT). In a 30-μl reaction, the gyrase complex and 0.5 μg of pBR322 relaxed DNA (Inspiralis, United Kingdom) were assembled on ice and incubated at 37°C for 30 min. The reaction was then stopped by adding an equal volume of stop solution (40% sucrose, 100 mM Tris-HCl [pH 7.5], 1 mM EDTA, 0.5 mg/ml bromophenol blue) and extracted by mixing with 30 μl chloroform-isoamyl alcohol (24:1). The samples were centrifuged at 14,000 × *g* for 1 min, and the aqueous phase was collected and loaded onto a 1% 1× Tris-acetate-EDTA (TAE) agarose gel. The DNA products were resolved by electrophoresis at 80 V for 120 min and visualized by UV irradiation. The images were then analyzed using ImageJ v.1.47 to obtain the intensity of the supercoiled DNA band. Gyrase with 1% dimethyl sulfoxide (DMSO) was used as the control. The percentage of supercoiled DNA (% SC) was normalized using samples with gyrase in the absence of inhibitor as the full supercoiling control and was plotted against PPG concentration. Data were fitted to the equation % SC = *ae^b^*^[PPG]^, where *a* and *b* are function parameters, using OriginLab Origin (Pro) v.2020b. The IC_50_ (concentration of PPG required for 50% supercoiling inhibition) was calculated from the fitted function IC_50_ (concentration of PPG required for 50% supercoiling inhibition).

### Microscale thermophoresis.

Microscale thermophoresis (MST) experiments were performed using a Monolith NT.115 instrument (NanoTemper). Proteins (*Pf*B and *Ec*B) were labeled with Red 2nd Generation labeling kits (NanoTemper) in which the fluorescent red dye was coupled with protein via NHS coupling. Labeled protein (100 nM) was mixed with PPG (Activate Scientific, dissolved in DMSO to a final concentration of 5%) and in MST buffer {240 mM NaP_i_ (pH 7.4), 274 mM NaCl, 54 mM KCl, 100 μM TCEP [Tris(2-carboxyethyl)phosphine hydrochloride], 0.05% Tween 20, 5% DMSO} and incubated in the dark for 15 min. The samples were applied onto capillaries (Monolith NT.115 MST Premium coated capillaries), and the fluorescence signal was measured using MO.Control software (Nanotemper). Initial fluorescence signal was used for binding analysis, and *K_d_* was calculated using 1:1 binding model. Results were baseline corrected and normalized for amplitude using MO.Control software (Nanotemper). The *K_d_* values were calculated based on three independent measurements. All experiments were performed at room temperature.

### ATPase activity.

The ATP hydrolysis reaction was performed as follows. Enzyme was incubated in the assay buffer (50 mM Tris-HCl [pH 7.5], 125 mM KCl, 1 mM EDTA, 5 mM MgCl_2_, 5 mM DTT, 10% glycerol) with test compounds at various concentrations, 1 mM ATP, and 10.5 nM linear pBR322 DNA for 30 min at room temperature. The protein concentrations used in the assay were as follows: 1 μM *Pf*B, 0.5 μM *Ec*A_2_*Pf*B_2_, and 100 nM *Ec*Gyr. A malachite green assay kit (Sigma-Aldrich) was used to measure the released phosphate in the reaction. In brief, malachite green reagent was added to the reaction mixture, and the mixture was further incubated for 10 min. The formed color was measured at 620 nm using a plate reader (SpectraMAX190; Molecular Devices). The amount of released phosphate was calculated based on a calibration curve covering a linear range of 50 to 250 pmol using eight concentrations, which was prepared for each measurement independently. Experiments were carried out in triplicate.

### Electrophoretic mobility shift assay.

DNA binding assays were carried out using electrophoretic mobility shift assay (EMSA) with 147 bp dsDNA (based on the preferred gyrase cleavage site from plasmid pBR322) (31). In brief, 20 nM DNA was mixed with 200 nM reconstituted complex (hybrid or *Ec*Gyr) or 1 μM *Pf*B in an assay buffer (30 mM Tris-HCl [pH 7.5], 75 mM KCl, 2 mM MgCl_2_, 6% glycerol, 1 mM DTT) and PPG for 1 h at room temperature. The samples were resolved in a 5% polyacrylamide gel in TBM buffer (90 mM Tris-borate, pH 7.5, 4 mM MgCl_2_) for 1 h at 80 V. The gel was stained with SYBR gold (Thermo) and visualized under UV light.

### Activity against Plasmodium falciparum.

Tests on parasites were performed by Anti-infectives Screening Core Services at NYU School of Medicine based on previously published protocol (44). P. falciparum strain 3D7 was used for all determinations. Using 96-well plates, 100 μl of P. falciparum growth medium (RPMI 1640, 25 mM HEPES, 0.1 mg/ml gentamicin, 0.05 mg/liter hypoxanthine [pH 6.75]), supplemented with 0.25% sodium bicarbonate and 0.5% Albumax II (Invitrogen), was added to each well. A 10 mM stock of PPG in DMSO was then serially diluted in triplicate. DMSO at the highest concentration used (0.5%) was tested in parallel as a control. Asynchronous P. falciparum in a 100-μl volume was then added to each well at 0.25% late-stage parasitemia with 5% hematocrit, as well as controls (medium, red blood cells, P. falciparum, or P. falciparum with 2 μM chloroquine). The plates were placed in a sealed chamber and maintained under atmospheric conditions of 5% oxygen, 5% carbon dioxide, and 90% nitrogen at 37°C for 96 h. To determine the cell viability, a SYBR green assay for rapid assessment was performed.

After incubation, plates were placed at −80°C for 24 h. The plates were then thawed at 37°C for 4 h. Once thawed, 100 μl of from each resuspended well was transferred into a black, 96-well plate. To each well, 100 μl of a solution containing 0.2 μl of SYBR green I nucleic acid dye (Molecular Probes) in 1 ml of lysis buffer (20 mM Tris-HCl [pH 7.5], 5 mM EDTA, 0.008% saponin, 0.08% Triton X-100) was added. The plates were covered in aluminum foil, followed by incubation on a plate shaker for 1 h at room temperature. Plates were analyzed on a fluorescence plate reader (Victor) at excitation and emission wavelengths of 485 and 530 nm, respectively. The counts were plotted against the logarithm of the PPG concentration, and curves were fitted by nonlinear regression. The IC_50_ was determined as the PPG concentration that produced 50% of the maximum counts from the drug-free controls. The results are the averages of triplicates.
