# Supplementary material for: Inhibitory Compounds Targeting Plasmodium falciparum Gyrase B
Source: Antimicrob Agents Chemother. 2021 Sep 17;65(10):e00267-21. doi: 10.1128/AAC.00267-21 (PMC8448092; doi:10.1128/AAC.00267-21)
Supplement: Supplemental file 1 — Supplemental material. Download AAC.00267-21-s0001.pdf, PDF file, 0.6 MB [file aac.00267-21-s0001.pdf]

Supplementary information

Inhibitory compounds targeting *Plasmodium falciparum* Gyrase B

Zuzanna Pakosz[a,b*], Ting-Yu Lin[c†*], Elizabeth Michalczyk[a,d], Soshichiro Nagano[c‡], Jonathan Gardiner Heddle[a,c#]

[a]Malopolska Centre of Biotechnology, Jagiellonian University, Krakow, 30-387, Poland

[b]Postgraduate School of Molecular Medicine, Medical University of Warsaw, Warsaw, 02-091, Poland

[c]Heddle Initiative Research Unit, RIKEN, Wako, Saitama, 351-0198, Japan

[d]Department of Cell Biochemistry, Faculty of Biochemistry, Biophysics and Biotechnology, Jagiellonian University, 30-387, Krakow, Poland

[†]Current Address: Malopolska Centre of Biotechnology, Krakow, 30-387, Poland

[‡]Current Address: Institute for Plant Physiology, Justus Liebig University Giessen, Giessen, D35390, Germany

[*]Equal contribution

Running title: Gyrase-targeting anti-malarial

[#]To whom correspondence should be addressed. Email: jonathan.heddle@uj.edu.pl

Supplementary methods

SDS PAGE

The purified proteins (2 µg) were subjected to a 10% SDS PAGE and resolved by electrophoresis at 180 V for 40 minutes. The protein bands were visualized by Coommassie Blue staining.

DNA cleavage assay

The DNA cleavage assay was performed in the same buffer conditions as for supercoiling but ATP was omitted and supercoiled DNA was used instead of relaxed DNA. *Ec*A_2*Pf*B_2 complex (150 nM) was incubated with 0.5 µg of supercoiled DNA in the absence/presence of inhibitors for 60 min at 37 °C. In order to monitor the production of linearized DNA, protein was removed by addition of 3 µl of 2% SDS and 1 µl of proteinase K (>700 U) followed by further incubation at 37 °C for 30 min. The DNA was then extracted and resolved in gel electrophoresis as described.

Supplementary Figure 1

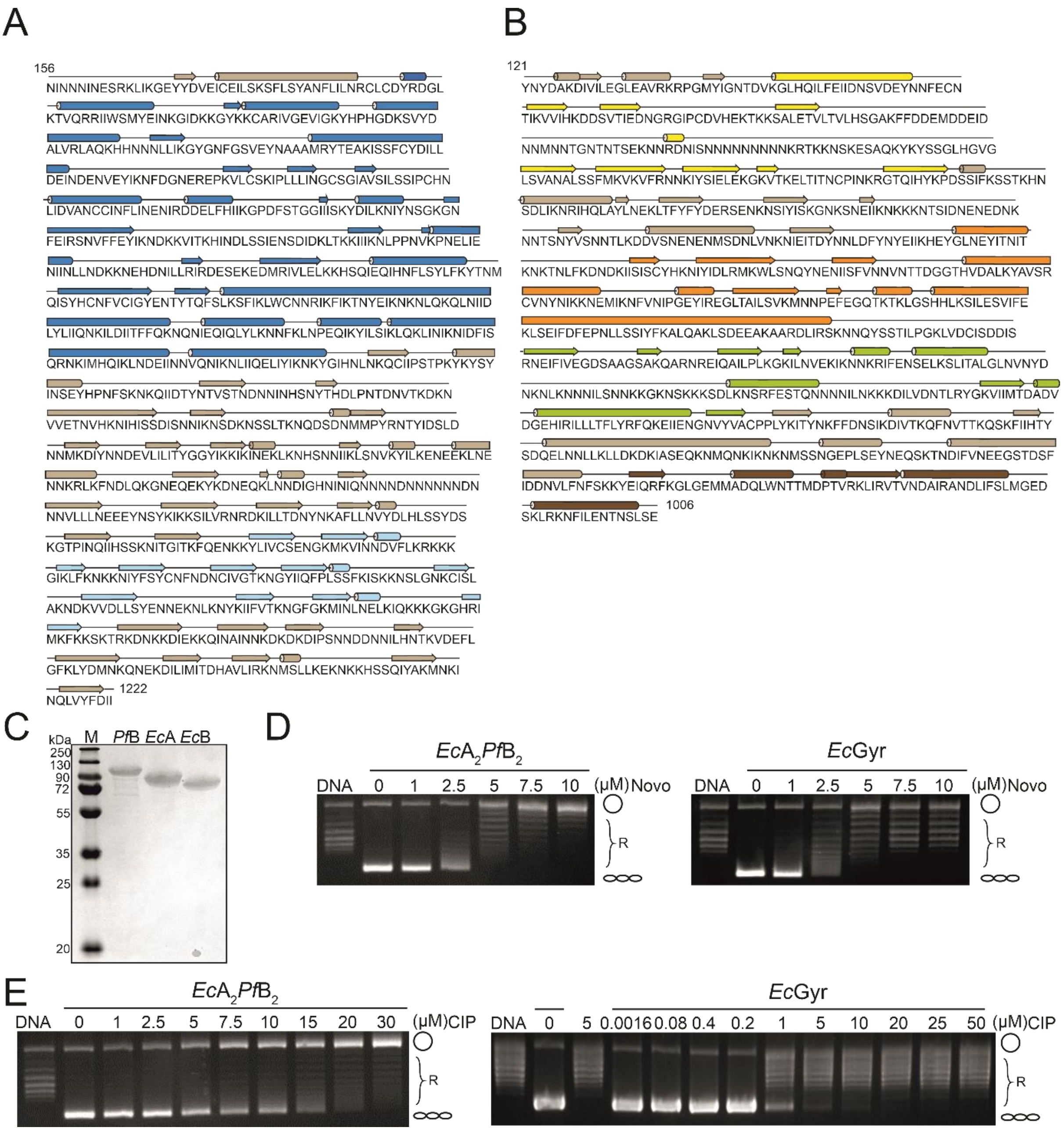

Fig. S1. Gyrase production and activities. (**A-B**) Amino acid sequences of mature forms of *Pf*A (**A**) and *Pf*B (**B**) with secondary structures illustrated above. (**C**) SDS-PAGE gel of purified recombinant proteins. (**D-E**) Effect of novobiocin (Novo) (**D**) and ciprofloxacin (CIP) (**E**) on DNA supercoiling activities of hybrid or *Ec*Gyr.

Supplementary Figure 2

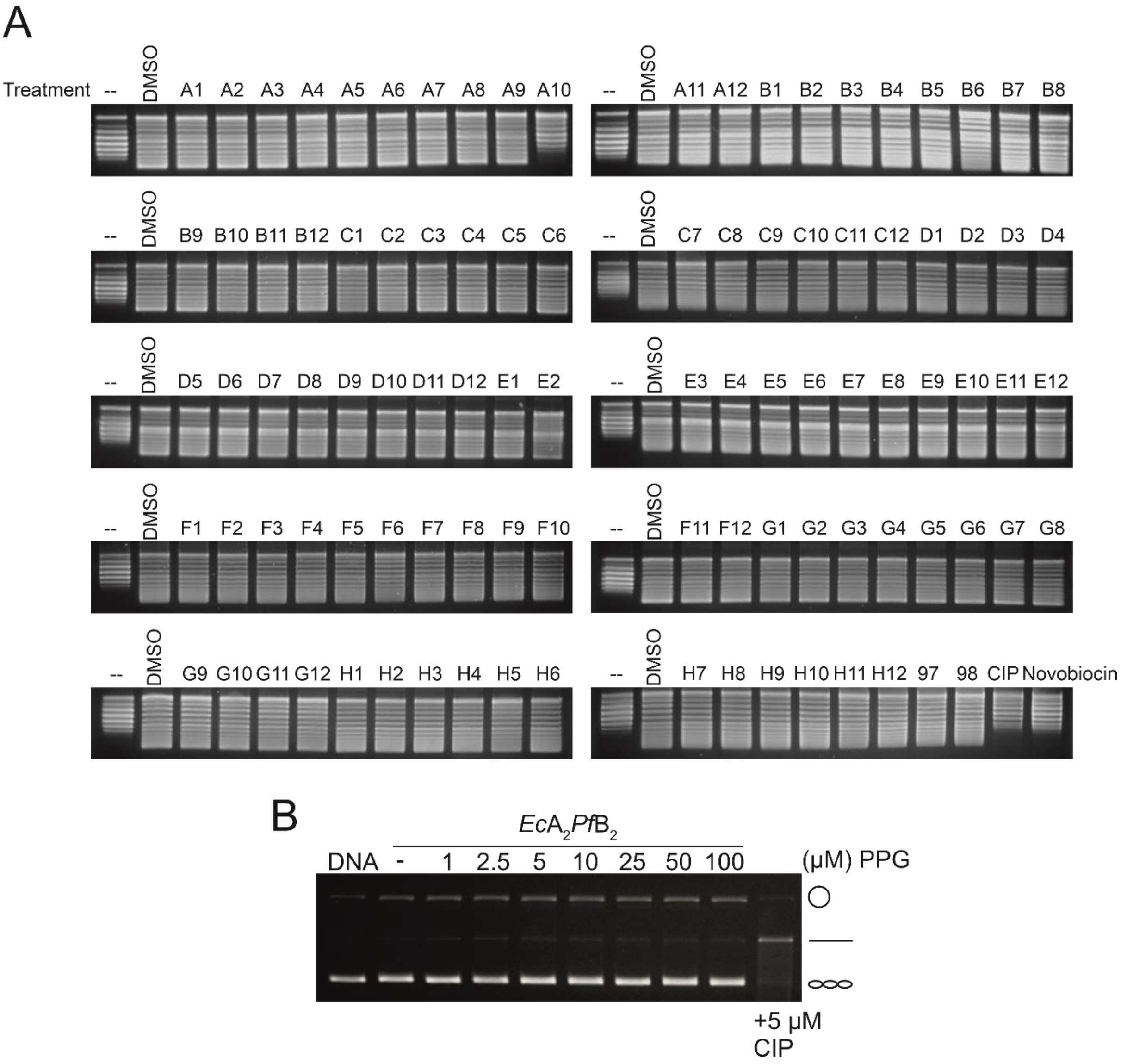

Fig. S2. The effect of small compounds on gyrase activities. (**A**) The small compound screening against the DNA supercoiling activity of hybrid complex. DMSO (5%) was included as the solvent control. A10 (PPG) and B6 (ARS) were chosen for further characterization. (**B**) *Ec*A$_2$*Pf*B$_2$-mediated DNA cleavage. Linearized DNA was resolved in the agarose gel and all forms of DNA were as indicated. Ciprofloxacin (5 µM) was used as a positive control in this assay.
